# Vasoactive Intestinal Peptide Tumor as the Cause of Persistent Diarrhea: A Diagnostic Challenge

**DOI:** 10.7759/cureus.29130

**Published:** 2022-09-13

**Authors:** Sara Lopes, Marta Alves, Pedro Rodrigues

**Affiliations:** 1 Endocrinology Department, Hospital de Braga, Braga, PRT

**Keywords:** somatostatin analogues, persistent diarrhea, vipoma, vasoactive intestinal peptide, functional neuroendocrine tumor

## Abstract

Although chronic diarrhea is frequent, some of its causes are rare, namely, neuroendocrine tumors (NETs). Due to their rarity and non-specific symptoms, such as diarrhea, they are often underdiagnosed.

An 80-year-old woman presented to the emergency department due to emesis and watery diarrhea. Blood tests showed acute kidney injury, hypokalemia, and metabolic acidosis. An abdominal computed tomography revealed a 51 mm pancreatic lesion. An endoscopic ultrasound-guided biopsy raised the hypothesis of a NET. The patient refused surgery and was lost to follow-up. At the eighth hospitalization, 11 months later, the suspicion of a vasoactive intestinal peptide tumor (VIPoma) was raised and confirmed by assessing the vasoactive intestinal peptide (VIP) levels (>100 pmol/L). Octreotide was started with the resolution of the symptoms. 68Ga-DOTANOC positron emission tomography/computed tomography excluded metastatic disease. After six months of octreotide therapy, the tumor shrunk 13 mm in maximum diameter. At the last follow-up, one year later, she remained asymptomatic.

The delayed diagnosis of VIPoma led to multiple life-threatening episodes. This case highlights the importance of considering all potential differential diagnoses of common symptoms such as diarrhea. Although VIPomas are rare, clinicians should be aware of this entity and suspect this diagnosis in patients with chronic diarrhea with a poor response to standard antidiarrheal agents. Somatostatin analogs should be promptly prescribed for symptom control and tumor progression prevention in patients who refuse surgery or have unresectable tumors. Tumor shrinkage might also be observed in these cases.

## Introduction

There are multiple possible causes of chronic diarrhea, some of them exceptionally rare, namely, neuroendocrine tumors (NETs) [1]. Vasoactive intestinal peptide tumors (VIPomas) are a type of NET with an incidence of 1 in 10 million per year. Due to their rarity and non-specific symptoms, such as chronic diarrhea, they are often underdiagnosed, leading to delayed treatment with potentially life-threatening consequences [2-5]. Additionally, there were no previous reports of tumor shrinkage using somatostatin analogs in VIPomas. Here, we report the case of a patient with a one-year history of watery diarrhea and multiple hospital admissions due to severe dehydration. She was ultimately diagnosed with a VIPoma, and octreotide was initiated with relevant tumor reduction.

## Case presentation

An 80-year-old woman went to the emergency department with a history of asthenia, emesis, and multiple daily episodes of watery non-bloody diarrhea initiated with a one-week duration. Her medical history included dyslipidemia and atrial fibrillation, being chronically medicated with simvastatin 20 mg daily and rivaroxaban 20 mg daily. She denied recent travel, dietary changes, sick contacts, the addition of new medications, ingestion of alcohol, smoking, or use of illicit drugs.

Blood tests revealed an acute kidney injury (urea nitrogen 267 mg/dL and creatinine 2.91 mg/dL), mild hyponatremia (Na^+^ 131 mmol/L), mild hypokalemia (K^+^ 3.1 mmol/L), and severe metabolic acidosis (arterial blood sampling: pH 7.052, pCO_2_ 21.3 mmHg, pO_2_ 103.9 mmHg, Cl^-^ 119 mmol/L, HCO^3-^ 5.8 mEq/L, lactic acid 0.5 mmol/L). C-reactive protein levels and leukocyte counts were within the normal range. During the hospital stay, an abdominal computed tomography (CT) revealed a 51 mm heterogeneous mass in the pancreatic body.

**Figure 1:**
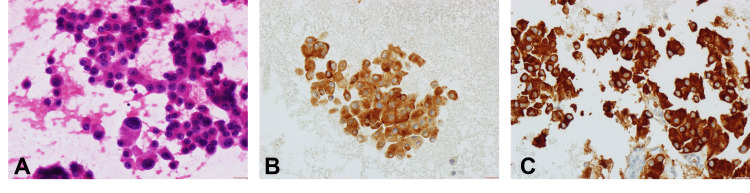
Cytologic features. Cells with round nuclei, prominent nucleoli, and granular cytoplasm. (A) Hematoxylin and eosin (400×) demonstrated positivity by immunohistochemistry for synaptophysin (B) and for MNF116 (C).

At discharge, the patient was referred to the general surgery outpatient department for further evaluation. Endoscopic ultrasound was performed and revealed a hypoechoic heterogeneous lesion with 50 × 45 mm in the pancreatic body without invasion of adjacent structures. Fine-needle aspiration showed cells with granular cytoplasm, round nuclei with prominent nucleoli, stained positive for synaptophysin and MNF116, and negative for CD56, setting the differential diagnosis of a NET or less likely of acinar cell carcinoma (Figure 1). Serum chromogranin A was mildly elevated (109.73 ng/mL; reference range (RR): <100 ng/mL). The patient refused surgery and was discharged from the general surgery outpatient clinic without further follow-up. Moreover, apart from loperamide, no further pharmacological treatment was suggested.

Over the following 11 months, the patient had eight visits to the emergency department owing to persistent watery diarrhea. Subsequently, she was hospitalized seven more times due to severe dehydration associated with acute kidney injury, metabolic acidosis, and hypokalemia. She presented a poor response to loperamide and reported daily diarrhea gradually worsening and persisting with fasting. No blood or mucus was found in the stools. She denied fever, episodes of constipation, abdominal bloating, or flushing. It is noteworthy that on the second hospital admission, stool samples for *Giardia lamblia* were positive, and the patient reported drinking water from an unsupervised source. The *Giardia *infection was treated, and she was discharged with the recommendation to suspend water consumption from the well. In subsequent hospitalizations, stool samples did not detect parasites or bacteria and the patient confirmed drinking exclusively bottled water.

**Figure 2:**
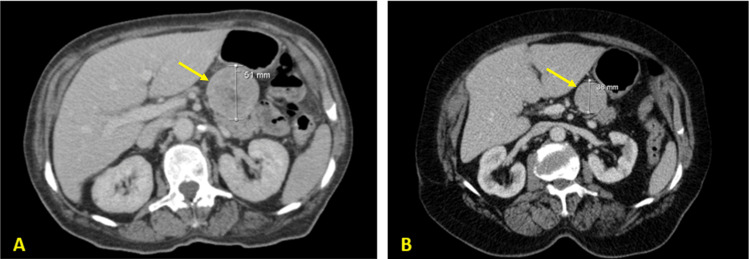
(A) Abdominal computed tomography (CT) scan at diagnosis (maximum diameter: 51 mm). (B) Abdominal CT scan after six months of octreotide long-acting release 20 mg 4/four weeks (maximum diameter: 38 mm).

At the eighth hospitalization, a VIPoma was suspected, and VIP levels were requested (>100 pmol/L; RR: <30 pmol/L). Given her clinical background, the elevated VIP levels, and the pancreatic lesion, the diagnosis of VIPoma was ultimately established. She was then referred to our department. Treatment with a long-acting somatostatin analog was initiated (octreotide long-acting release, 20 mg every four weeks) with the resolution of diarrhea and metabolic disturbances. A 68Ga-DOTANOC PET/CT was performed showing no evidence of metastatic disease. Six months after starting octreotide, the maximum diameter of the tumor had reduced by approximately 25% (from 51 mm to 38 mm) (Figure 2). At the last follow-up, five months later (and 11 months after instituting the somatostatin analog), the patient remained asymptomatic.

## Discussion

This case highlights the importance of recognizing this rare entity because a delay in treatment can lead to multiple hospital admissions, poor quality of life, and life-threatening situations. VIP is a neuropeptide that activates cyclic adenosine monophosphate and adenylate cyclase in intestinal cells. This interaction leads to reduced absorption in the small intestine and increased bowel motility and secretion of electrolytes and fluids. Ultimately, excessive VIP secretion induces metabolic acidosis and the classically called WDHA syndrome (watery diarrhea, hypokalemia, and achlorhydria). Although this neuropeptide also inhibits gastric acid secretion, achlorhydria is not an obligatory finding, and hypochlorhydria, or even normal acid secretion, can occur [4,5]. This case also reinforces the pertinent role of somatostatin analogs as an alternative to surgery for symptom control in patients with unresectable masses and in patients who cannot undergo surgery or refuse this procedure [2-5,6-10]. In fact, currently, they remain the suggested first-line medical treatment [2-4,10,11]. VIPomas typically have somatostatin receptors on their surface, as is the case for most NETs. Thus, somatostatin analogs will attach to these receptors, inhibiting hormone secretion from these tumors. Notwithstanding, follow-up of patients under these drugs is essential, as additional modalities of treatment may be required to limit disease progression and for further symptom control. Nevertheless, although not always an effective procedure, surgical resection remains the only curative option in localized disease without metastasis, which was our case [2,3,8,10,12]. However, this does not reflect the majority of patients; as many as 80% present with metastasis at diagnosis [2,4,11]. In unresectable tumors, surgical cytoreduction has not been proven advantageous. Therefore, therapeutic alternatives have been proposed in the last decades: somatostatin analogs, systemic chemotherapy, interferon-alpha, radiofrequency ablation, and embolization [2-4,6,7,12,13]. More recently, sunitinib and peptide receptor radionuclide therapy (PRRT) with radiolabeled somatostatin analogs (177Lu-DOTATATE) have also been described as viable options [2-4,8,14]. Additionally, evidence shows that sunitinib and chemotherapy are adequate substitutes for surgery as both combine anti-proliferative with anti-secretory effects [8,14]. Contrastingly, despite their well-established role in symptom control and their known effect on tumor stabilization, evidence is scarce regarding the impact of somatostatin analogs on lesions’ shrinkage [9]. Indeed, there are few published cases of reduction of gastroenteropancreatic NETs reduction with these drugs, and, to the best of our knowledge, there were no previous reports specifically of non-metastasized VIPomas [9,15]. Nonetheless, there are available data regarding metastasis shrinkage in this type of functional NET [16,17]. Therefore, these medications should not only be considered a tool for controlling hormone-related symptoms and tumor growth but also as having the potential to reduce tumoral size.

Nevertheless, for clinicians to prescribe these potentially life-saving medications and prevent numerous complications, they need to promptly diagnose this disease, which was not the case here. Notwithstanding the cytological report raising the possibility of a NET, the patient was discharged after refusing surgery, without being offered an effective treatment alternative or referral to other specialties. After being lost to follow-up, the patient had multiple visits to the emergency department. It could be argued the diagnosis should have been made sooner. History of *Giardia lamblia* and consumption of unsupervised water possibly acted as confounding factors; however, these two situations were uncovered and resolved on the second hospitalization. We believe the most preponderant factor for the delay was the fact that, in general, physicians do not have NETs as a differential diagnosis right away due to their rarity and non-specific symptoms, such as diarrhea. Indeed, an international survey found a mean 52-month delay in the diagnosis of NETs and patients must see an average of six different clinicians before they get the correct diagnosis [18]. Our patient also had a significant time gap between symptom onset and diagnosis. During 11 months, she went nine times to the emergency rooms of two different hospitals, was hospitalized eight times, and was evaluated by multiple clinicians before the VIPoma was detected. However, it should be said that, although traditionally seen as a rare entity, the incidence of NETs has been increasing worldwide, with some studies reporting a prevalence as high as 6.6 cases per 100,000 people [19]. The usual clinical manifestations of VIPomas are persistent watery diarrhea, acidosis, hypokalemia, and achlorhydria due to hypersecretion of VIP, as previously mentioned [2-4,10,11]. Biochemical diagnosis is made when VIP is markedly elevated (>200 pg/mL; 148 pmol/L). Nevertheless, it should be suspected in patients with unexplained watery diarrhea, a pancreatic mass, and VIP >75 pg/mL (>55 pmol/L). After high VIP levels are demonstrated, the majority of VIPomas can be localized by CT scan or endoscopic ultrasound, and metastatic disease should be investigated with a 68Ga-DOTANOC PET/CT [2-4,10,11,20]. In most cases, the primary tumor is found in the pancreas (75-90%), as was the case in our patient [4,5].

## Conclusions

Although VIPomas are rare, clinicians should be aware of this potentially life-threatening entity and suspect this diagnosis in patients with chronic watery diarrhea that persists with fasting and has a poor response to standard antidiarrheal agents. Somatostatin analogs should be promptly prescribed for symptom control and tumor progression prevention in patients who refuse surgery or have unresectable tumors. Tumor or metastasis shrinkage might also be observed in these cases.
